# Fixation Improvement through Biofeedback Rehabilitation in Stargardt Disease

**DOI:** 10.1155/2016/4264829

**Published:** 2016-04-24

**Authors:** G. Scuderi, F. Verboschi, D. Domanico, L. Spadea

**Affiliations:** ^1^Ophthalmology Unit, NESMOS Department, Sant'Andrea Hospital, “Sapienza” University of Rome, Via di Grottarossa 1035-1039, 00189 Rome, Italy; ^2^Department of Ophthalmology, A. Fiorini Hospital, “Sapienza” University of Rome, Via Firenze, 04019 Terracina, Italy

## Abstract

Stargardt disease is the most common hereditary macular degeneration in juveniles. It is characterized by macular dystrophy associated with loss of central vision in the first or second decade of life, a “beaten-metal” appearance in the fovea or parafoveal region, yellowish flecks around the macula or in posterior area of the retina, progressive atrophy of the bilateral foveal retinal pigment epithelium, and the “dark choroid” sign on fundus fluorescein angiography in most cases. We report a case of Stargardt disease in a 26-year-old Caucasian female submitted to rehabilitative training with microperimetry MP-1 to find a new preferred retinal locus (PRL) and to train her to better her quality of life. Best corrected visual acuity, mean retinal sensitivity, fixation, bivariate contour ellipse area, and speed reading were evaluated before and after the training and results were discussed.

## 1. Introduction

Stargardt disease is an autosomal recessive condition commonly seen in early adulthood characterized by the presence of classical yellow subretinal flecks at the posterior pole. The macular changes can range from fine granularity to beaten bronze metal appearance to geographic atrophy. Fishman et al. analysed ninety-five patients with Stargardt macular dystrophy for visual loss with age and they showed that the probability of maintaining a visual acuity of 20/40 or better in at least one eye was 52% by age 19, 32% by age 29, and 22% by age 39; furthermore in the population studied by Fishman, once a patient's visual acuity dropped below 20/40, it tended to decrease rapidly and stabilize at 20/200 [1]. Visual acuity changes in Stargardt disease from the first stage to the last and in a majority of cases with stage 1 were more likely to maintain 20/200 or better visual acuity in at least one eye when compared with patients with stage 2/stage 2-3 Stargardt disease [2].

We report a case of Stargardt disease in a 26-year-old Caucasian female that presented to us complaining of visual impairment in both eyes for 3 years.

Presentation, clinical findings, and morphological changes are discussed. After ascertaining the diagnosis, the patient underwent a rehabilitation protocol by biofeedback training with MP-1 trying to find a new preferred retinal locus (PRL) and improve her quality of life. The rehabilitation protocol to which the patient was subjected was showed and results were discussed.

## 2. Case Presentation

A Caucasian female aged 26 years complained of a decrease in visual acuity progressive for 3 years. There was no previous ophthalmic history and her general health was good. The patient denied having eye pain, redness, photophobia, or irritation. She was not taking any medications and denied any medications allergies. The best corrected visual acuity (BCVA) was 20/200 in the right eye (RE) and 20/100 in the left eye (LE). The examination of the anterior segment in both eyes was unremarkable: the bulbar conjunctiva was white and transparent, anterior chamber clear, and cornea transparent. Furthermore, pupils were normal in size or shape and reactive to light, and no lens opacification was seen. The fundus examination revealed the existence of bilateral atrophy of the foveal retinal pigment epithelium (RPE) and photoreceptors and yellow-orange flecks distributed around the macula and the midretinal periphery. The vitreous is transparent; the examination of the extreme peripheral retina is normal.

The photographs in autofluorescence by Fundus Autofluorescence (FAF; HRA 2, Heidelberg Engineering) described a granular appearance of macula with alternating of hypoautofluorescent (alteration of the pigment epithelium) and hyperautofluorescent (lipofuscin deposition) dots. These lesions are accompanied by flavimaculatus spots. The fluorescein angiography (FA; HRA 2, Heidelberg Engineering) confirmed the presence of macular atrophy accompanied by a reorganization of the pigment epithelium. There is an oval shaped window defect centered on the fovea. Optical coherence tomography (OCT; Spectralis, Heidelberg Tomography) showed a bilateral macular atrophy, with disappearance of the ellipsoid line. The electroretinogram and electrooculogram were normal. All these diagnostic tests concluded the diagnosis of Stargardt disease type 1.

We decided to submit the patient to rehabilitation program to improve her quality of life. Then a written informed consent was obtained by her. The rehabilitation protocol consisted of a 25-item questionnaire (National Institute Visual Functioning Questionnaire, NEI-VFQ-25) to test her quality of life, reading speed test (words/minute) calculated by trial lenses in addition to reading appropriate for the age, on a text in Times New Roman 18 printing body, calculating the number of words per minute, then microperimetry and fixation test with microperimeter MP-1, and then 10 training sessions with MP-1 acoustic target biofeedback.

Microperimetry and fixation test were performed with MP-1 microperimeter (NIDEK Technologies Srl, Padova, Italy) using the automated program, the threshold test of 4-2 strategy, and a 1° single cross fixation target. However, at the beginning of the study the size was enlarged to a 2° single cross fixation target when the patient was not able to see the 1° single cross fixation target.

Fixation stability was quantified according to Fujii classification and also by calculating the bivariate contour ellipse area (BCEA) encompassing 68% of fixation points based on collected fixation data after 30 seconds. The patient had a lot of eccentric and unstable loci of fixation around the fovea and in fact the initial BCEA was very large because the eccentricity of the PRL from the fovea ranged from 1.8° to 8.6°. We have also calculated retinal threshold sensitivity measured in all eyes using the Goldmann III target (round shape with a white background) with stimulus intensity ranging from 0 to 20 dB. Stimulus presentation time was 200 ms. After microperimetry we have chosen the preferred retinal locus (PRL) more suitable to train in an area 3° above the fovea in both eyes, which is an eccentric point compared to the fovea but in which there was greater retinal sensitivity, and we trained it: the patient was asked to move their eyes according to audio feedback which advised her whether she was getting closer to the desired final fixation position.

**Figure 1:**
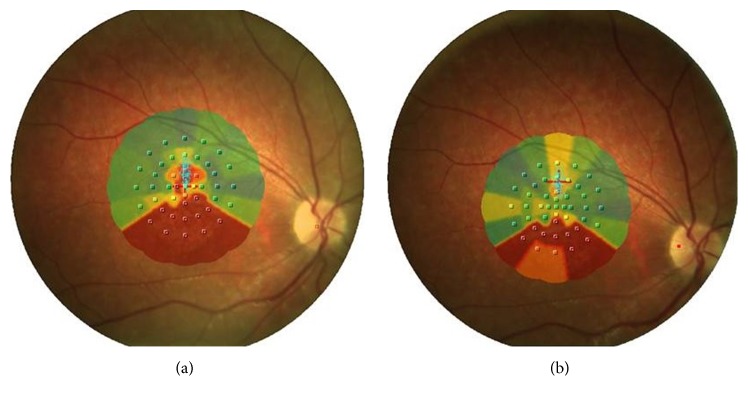
(a) Interpolated microperimetric map of RE before rehabilitation at zero time in which we see the image of foveal atrophy that does not allow a good fixation of the target (red cross). (b) Interpolated microperimetric map at the end of rehabilitation training after 12 months in which an improvement of fixation stability through the identification of TRL was achieved.

The rehabilitation protocol consisted of 10 training sessions of 10 minutes for each eye, performed once a week using the MP-1 acoustic target biofeedback examination. All the procedures were followed on a monitor. The training was repeated after 3 and 6 months and after one year we have evaluated results. At the end of the rehabilitation, the 25-item questionnaire (National Institute Visual Functioning Questionnaire, NEI-VFQ-25), the assessment of distant and near visual acuity, reading speed test, and fixation and microperimetry tests were repeated. BCVA was maintained till the follow-up after a year. The mean retinal sensitivities before and after 1 year in the RE were 2.9 dB and 3.7 dB, respectively, while in the LE they were 3.5 dB and 4.8 dB, respectively. She has demonstrated an improved fixation that increased from being unstable to relatively unstable according to Fujii classification in both eyes; BCEA became from 3.52 deg^2^ to 1.87 deg^2^ in the RE while in LE it became from 3.27 deg^2^ to 1.54 deg^2^ and this result demonstrated an objective improvement of fixation (Figure 1). The reading speed has improved from 18 words/minute to 27 words/minute and her score in the NEI-VFQ-25 was higher than before the rehabilitation training, from 37.4 ± 9.8 to 56.2 ± 3.2. The patient is declared summarily satisfied with the visual performance obtained with the exercise of biofeedback.

## 3. Discussion

Stargardt disease, first described in 1909, is an autosomal recessive macular dystrophy. The majority of people affected by the disease present with uncorrectable, decreased visual acuity during their teenage years, which most often progresses to legal blindness. To date, there is no approved intervention. Stargardt disease is marked by premature accumulation of lipofuscin in the retinal pigment epithelium (RPE), degeneration of the neuroretina, and subsequent loss of vision. The condition results from mutations in the ATP-binding cassette, subfamily A, member 4 (*ABCA4*) gene A photoreceptor cell-specific ATP-binding transporter gene (ABCR) that is mutated in recessive Stargardt macular dystrophy [3], which encodes a transmembrane flippase localized in photoreceptor outer segments. The flippase transports the phosphatidyl-ethanolamine-retinaldehyde Schiff base between the cytosol and the cytoplasmic disk surfaces [4]. Mutations in* ABCA4* also result in retinitis pigmentosa and cone-rod dystrophy and have been linked to age-related macular degeneration (AMD) [5, 6]. Biofeedback techniques applied to vision are still being studied in both their methodological and physiological aspects. Some authors [7–10] have proposed different visual rehabilitation techniques and instruments using biofeedback strategies starting from basic systems like Accommotrac Vision Trainer or Improved Biofeedback Integrated System (IBIS) devices, merging to more complex instruments as the fundus related MP-1 microperimeter (NIDEK Technologies Srl, Padova, Italy) and MAIA microperimeter (CenterVue, Padova, Italy) [11].

Visual rehabilitation with biofeedback is a therapeutic approach that has been applied in different ocular pathologies characterized by visual deterioration and loss of fixation stability: nystagmus, AMD, glaucoma, anisometropia, amblyopia, retinitis pigmentosa, oculocutaneous albinism, myopic maculopathy, vitelliform dystrophy, posttraumatic macular scar, and cone dystrophy.

Olivo et al. demonstrated that Stargardt disease patients showed a significant gray matter (GM) loss bilaterally in the occipital cortices, extending into the right precuneus, and in the frontoorbital cortices. At tract-based spatial statistics (TBSS), significant reductions in fractional anisotropy were detected throughout large regions in the supratentorial white matter (WM), more pronounced in the posterior areas. Gray matter volume correlated directly with mean visual sensitivity in the right middle frontal and left calcarine gyri and inversely with retinal thickness in the left supramarginal gyrus [12].

In this case report we wanted to find a new fovea (PRL) and turn it into a trained retinal locus (TRL) [13]. Our results showed that the new PRL (TRL) increased fixation stability as well as retinal sensitivity and reading speed.

Improvement through biofeedback training in patients who suffer from macular disease either remaining stable or worsening, where the traditional treatment cannot offer further results, is of interest and well worthy of attention. The reasons for this improvement are probably due to the fact that we trained a “retinal motor” PRL, with appropriate retinal sensitivity, so as to increase the number of correct fixation saccades and rereference the oculomotor system.

Andrade has shown that patients are usually unaware of their scotoma because when the retina is damaged by a local lesion (induced scotoma), the cortical neurons driven by stimuli originating in this region do not remain inactive but become selective to stimuli originating in other parts of the retina. This process occurs in two distinct steps, each with its own time scale: (a) a fast redistribution of receptive fields (RFs) in the area of the lesion and (b) a long-term reorganization that leads to the final RF configuration. Cortical neurons located in the retinotopic position corresponding to the scotoma receive some degree of activity from the unimpaired neurons in the area surrounding the lesion [14]. This is the result of cortical plasticity. Sound perception increases the conscious attention of the patient [15, 16], thereby facilitating the lock-in of the visual target and increasing the permanence time of the target itself on the retina. This mechanism probably facilitates stimuli transmission between intraretinal neurons as well as between the retina and brain, where the highest degree of stimuli processing takes place, thereby supporting a “remapping phenomenon.” This case report has demonstrated that microperimetric biofeedback is a simple and low cost training which has important effects on the quality of life of this patient and it has an extremely low rate of complications.
